# The use of validated and nonvalidated surrogate endpoints in two European Medicines Agency expedited approval pathways: A cross-sectional study of products authorised 2011–2018

**DOI:** 10.1371/journal.pmed.1002873

**Published:** 2019-09-10

**Authors:** Catherine Schuster Bruce, Petra Brhlikova, Joseph Heath, Patricia McGettigan

**Affiliations:** 1 William Harvey Research Institute, Queen Mary University of London, Charterhouse Square, London, United Kingdom; 2 The Institute of Health and Society, Newcastle University, Newcastle upon Tyne, United Kingdom; 3 Greater Manchester Mental Health NHS Foundation Trust, Prestwich, Manchester, United Kingdom; Harvard University, Brigham and Women's Hospital, UNITED STATES

## Abstract

**Background:**

In situations of unmet medical need or in the interests of public health, expedited approval pathways, including conditional marketing authorisation (CMA) and accelerated assessment (AA), speed up European Medicines Agency (EMA) marketing authorisation recommendations for medicinal products. CMAs are based on incomplete benefit-risk assessment data and authorisation remains conditional until regulator-imposed confirmatory postmarketing measures are fulfilled. For products undergoing AA, complete safety and efficacy data should be available, and postauthorisation measures may include only standard requirements of risk management and pharmacovigilance plans. In the pivotal trials supporting products assessed by expedited pathways, surrogate endpoints reduce drug development time compared with waiting for the intended clinical outcomes. Whether surrogate endpoints supporting products authorised through CMA and AA pathways reliably predict clinical benefits of therapy has not been studied systematically. Our objectives were to determine the extent to which surrogate endpoints are used and to assess whether their validity had been confirmed according to published hierarchies.

**Methods and findings:**

We used European Public Assessment Reports (EPARs) to identify the primary endpoints in the pivotal trials supporting products authorised through CMA or AA pathways during January 1, 2011 to December 31, 2018. We excluded products that were vaccines, topical, reversal, or bleeding prophylactic agents or withdrawn within the study time frame. Where pivotal trials reported surrogate endpoints, we conducted PubMed searches for evidence of validity for predicting clinical outcomes. We used 2 published hierarchies to assess validity level. Surrogates with randomised controlled trials supporting the surrogate-clinical outcome relationship were rated as ‘validated’. Fifty-one products met the inclusion criteria; 26 underwent CMAs, and 25 underwent AAs. Overall, 26 products were for oncology indications, 10 for infections, 8 for genetic disorders, and 7 for other systems disorders. Five products (10%), all AAs, were authorised based on pivotal trials reporting clinical outcomes, and 46 (90%) were authorised based on surrogate endpoints. No studies were identified that validated the surrogate endpoints. Among a total of 49 products with surrogate endpoints reported, most were rated according to the published hierarchies as being ‘reasonably likely’ (*n* = 30; 61%) or of having ‘biological plausibility’ (*n =* 46; 94%) to predict clinical outcomes. EPARs did not consistently explain the nature of the pivotal trial endpoints supporting authorisations, whether surrogate endpoints were validated or not, or describe the endpoints to be reported in the confirmatory postmarketing studies. Our study has limitations: we may have overlooked relevant validation studies; the findings apply to 2 expedited pathways and may not be generalisable to products authorised through the standard assessment pathway.

**Conclusions:**

The pivotal trial evidence supporting marketing authorisations for products granted CMA or AA was based dominantly on nonvalidated surrogate endpoints. EPARs and summary product characteristic documents, including patient information leaflets, need to state consistently the nature and limitations of endpoints in pivotal trials supporting expedited authorisations so that prescribers and patients appreciate shortcomings in the evidence about actual clinical benefit. For products supported by nonvalidated surrogate endpoints, postauthorisation measures to confirm clinical benefit need to be imposed by the regulator on the marketing authorisation holders.

## Author summary

**Why was this study done?:**

- The approval of new pharmaceutical products addressing an unmet need or of public interest may be speeded up if medicines regulators agree to assess them through expedited assessment pathways.
- Pharmaceutical product development may be speeded up by using surrogate (substitute) endpoints (for example, based on a blood test or a radiological change) to measure benefit in the pivotal (main) trials assessed by regulators rather than waiting on the intended clinical outcome (benefits in how patients feel, function, or how long they survive).
- It is not known how often regulatory approval is based on a surrogate endpoint instead of a clinical outcome.
- It is not known how accurately the surrogate endpoints used in pivotal trials correspond to improvement in clinical outcomes.

**What did the researchers do and find?:**

- We examined endpoints supporting regulatory approval of products assessed through 2 expedited assessment pathways, conditional marketing authorisation (CMA; 26 products) and accelerated assessment (AA; 25 products) by the European Medicines Agency (EMA) between 2011 and 2018.
- Where surrogate endpoints were used, we searched the academic literature for studies demonstrating that the surrogate reliably reflected a clinical outcome.
- Most of the expedited approvals studied (46/51; 90%) were based on surrogate endpoints, and none of these has (yet) been shown to reliably predict clinical outcomes.
- The information provided by the regulator for prescribers and patients did not consistently explain that the approval for the product was based on trials that reported surrogate endpoints rather than clinical outcomes.

**What do these findings mean?:**

- For the majority of the products studied, we do not know whether they will provide their intended clinical benefit for patients.
- When nonvalidated surrogate endpoints are used in pivotal trials, a requirement for additional studies confirming the clinical benefit to patients needs to be imposed by the regulator on the manufacturing authorisation holder responsible for the product.
- It would be of great help for patients and doctors if the product information provided by the regulator clearly stated whether surrogate endpoints or actual clinical outcomes were reported in the pivotal trial/s for the product, the validity of a surrogate endpoint (when used) as a reflection of the intended clinical outcome, and whether follow-up studies demonstrating clinical benefit were required for products approved on the basis of surrogate endpoints in the pivotal trials.
- A limitation of our study is that it applies to products approved through 2 expedited pathways; we do not know to what extent other types of regulatory approval are based on surrogate endpoints.

## Introduction

The European Medicines Agency (EMA) is the central regulatory body providing recommendations on the authorisation (approval) of new medicinal products in the European Union (EU) [1]. In situations of ‘unmet medical need’ and/or ‘in the interests of public health’ [2], conditional marketing authorisation (CMA) and accelerated assessment (AA) pathways provide expedited routes to EMA authorisation recommendations.

CMAs are based on incomplete benefit-risk assessment data, and the authorisation is conditional on completing regulator-imposed postmarketing measures ‘with a view to providing comprehensive data confirming that the benefit-risk balance is positive’ [3,4]. Products undergoing AA have a shortened regulatory assessment period compared with standard assessments. They require complete safety and efficacy data to be available and may have postauthorisation conditions or restrictions, including obligations to conduct postauthorisation safety studies (PASSs) described in their risk management plans (RMPs) (Table 1) [5].

**Table 1:** Expedited pathways for early EMA marketing approval [3–6].

	CMA	AA
**Introduced**	2006	2004
**Purpose**	Earlier authorisation of products for patients with unmet medical needs on the basis of less complete clinical data	Reduction of assessment time for marketing authorisation applications to 150 days or less (compared with standard 210 days)
**Eligibility Criteria**	Products for which the benefit-risk balance is such that expedited access offsets the limitations of an incomplete data set, i.e., products that potentially address an ‘unmet medical need’	Products of major interest from a public health perspective—typically, that the products address, to a significant extent, the unmet medical needs that maintain/improve the health of society, i.e., introducing new methods of therapy or improving existing ones
**Evidence Requirements**	Safety and efficacy evidence profile incomplete at time of authorisation	Full safety and efficacy evidence profile available at time of authorisation
**Postmarketing Requirements**	Authorisations are subject to specific obligations	Obligations may apply
**Specific Obligations**	Yes—ensure comprehensive evidence is generated after authorisation within an agreed time frame	No

**Abbreviations:** AA, accelerated assessment; CMA, conditional marketing authorisation; EMA, European Medicines Agency.

Pivotal trials or ‘main studies/trials’ that provide benefit-risk data to support authorisation recommendations may be based on ‘clinically meaningful’/‘clinical’ endpoints (or outcomes) or surrogate endpoints. A clinical endpoint/outcome is described as ‘a characteristic or variable that reflects how patients feel, function or how long they survive’ [7,8]. Surrogate endpoints are biomarkers or intermediate endpoints intended to substitute for and predict a clinical outcome [7,8]. They may be validated or nonvalidated. A validated surrogate endpoint is one in which the treatment effect on the surrogate corresponds to the effect on the intended clinical outcome and hierarchies of validity are described in the literature [7,8]. For example, blood pressure has been shown to predict mortality from cardiovascular disease. Nonvalidated surrogate endpoints are not supported by evidence demonstrating that they reliably predict clinical outcomes. For example, despite its widespread use in studies of oncology therapies, the surrogate endpoint of progression-free survival (PFS), a composite of time to tumour progression or death, has been found to have highly variable correlation with the desired clinical outcome of overall survival [9–11]. Consequently, products recommended for authorisation based on nonvalidated surrogate endpoints may not reliably provide the intended clinical benefits for patients [8,12–14].

Both validated and nonvalidated surrogate endpoints have been accepted in place of clinical endpoints by medicines regulators, including for products recommended for approval through expedited pathways. Surrogate endpoints reduce drug development time, permitting new discovery benefits to reach patients faster than waiting on clinical endpoints, and they may be practically and/or ethically preferable [6]. EMA has reported CMA as advancing authorisation by an average of 4 years, but in the United States, regulatory acceptance of surrogate outcomes in oncology trials has been estimated to save just 11 months of drug development time, on average, compared with waiting on meaningful clinical outcomes [15,16]. The ‘Accelerated Approval Pathway’ enables products to enter US markets based on clinical trials with surrogate endpoints that are only required to be ‘reasonably likely’ to predict clinical outcomes [17,18].

In the EU, pivotal trials supporting marketing authorisation recommendations through CMA and AA pathways use both clinical and surrogate endpoints. In both cases and in common with other approval pathways, postmarketing requirements include risk management and pharmacovigilance plans. Products granted CMA are subject to additional specific obligations imposed by the regulator and described in the summary of product characteristics (SpC) document, Section E of Annex II, requiring the marketing authorisation holder to collect additional data to complete the benefit-risk profile. Where a product authorisation recommendation has been based on a surrogate endpoint, specific obligations could potentially require postmarketing studies demonstrating the intended clinical benefit or validating the endpoints in the case of nonvalidated surrogates. For products undergoing AA on the basis of surrogate endpoints, the true clinical benefit of the products approved for marketing may never be established because there is no consistent requirement for confirmatory postmarketing studies.

The reliance on surrogate endpoints and the extent to which they are validated have not been studied systematically. In this cross-sectional study, our objective was to examine whether authorisations granted in Europe through 2 expedited pathways, CMA and AA, were based on clinical or surrogate endpoints. Where surrogate endpoints were used, we assessed whether they were validated or nonvalidated and determined whether postauthorisation measures to confirm clinical outcomes had been required by the regulator.

## Methods

We used the EMA website search tool to identify products granted CMA between January 1, 2011, and December 31, 2018 [19]. We obtained the names of products authorised via the AA pathway during January 1, 2011, and December 31, 2017, through a request (via Regulation [EC] No. 1049/2001) to EMA; for January 1, 2018–December 31, 2018, we obtained the names from the EMA annual report for 2018 [20]. We excluded the following products: vaccines; reversal agents, e.g., idarucizimub (Praxbind, AA), for anticoagulant-associated bleeding; topical agents, e.g., cenegermin (Oxervate, AA), for ophthalmic keratitis; those without pivotal trials, e.g., ketoconazole (Ketoconazole HRA, AA), for Cushing’s disease; bleeding prophylaxis agents, e.g., human coagulation Factor X (Coagadex, AA), for Factor X deficiency-associated bleeding prevention; and those no longer authorised at time of data collection, e.g., boceprevir (Victrelis), for chronic hepatitis C infection, withdrawn during 2018 at the request of the marketing authorisation holder. We also excluded products authorised via a third expedited pathway, exceptional circumstances, products for which comprehensive data on efficacy and safety cannot be provided ‘because the condition to be treated is rare or because collection of full information is not possible or is unethical’ [21]. These products are unlikely ever to have a full dataset as is expected for products granted CMA or AA.

Noting that Banzi and colleagues had examined CMAs for the period from January 2006 to June 2015, we opted to minimise overlap by studying approvals from 2011 onwards because, by our study commencement, many products granted CMA prior to 2011 had achieved full approval owing to completion of specific obligations [6]. Our study protocol was prespecified in alignment with the objectives and comprised the steps described in the subsequent paragraphs (S1 Text).

We used European Public Assessment Reports (EPARs) to determine the endpoint(s) used in the pivotal trial(s) supporting product authorisations, the rationale for the endpoint choice, and specific obligations in the case of CMAs and postauthorisation conditions or restrictions in the case of products undergoing AA. We focused on the pivotal (or main) trials described in EPAR Section 2.5.2 because these had provided the main evidence basis for authorisation. We checked whether surrogate endpoints had an EMA qualification opinion on their acceptability or context of use for regulatory decision-making [22].

For products granted CMA, the authorisation date on the search tool and in the EPAR did not always concur; we used the search tool date. We used the SpCs to cross-check (December 2018) authorisation status and specific obligations (Annex ll, Section E) of products initially granted CMA and postauthorisation requirements or restrictions applying to products that had AA (Annex ll, Section D). Products undergoing CMA that were also granted AA are reported here as CMA because a full dataset was unlikely to exist at the time of authorisation.

We used the description of Fleming and Powers (F&P) as the basis for identifying validation studies: ‘validating a surrogate endpoint requires providing an evidence-based justification, often from randomised controlled clinical trials, that achievement of substantial effects on the surrogate endpoint reliably predicts achievement of clinically important effects on a clinically meaningful endpoint’ in conjunction with the description of Ciani and colleagues (‘Ciani’) [7,8].

We conducted PubMed searches to investigate whether surrogate endpoints had validation studies. The PubMed search terms were as follows: [‘endpoint’] and [validat* surrogate outcome OR validat* surrogate endpoint OR validat* surrogate end-point] and [‘indication’] (i.e., the ‘therapeutic indication’ described in ‘product information’ provided on the EMA website). Following reviewer advice, we repeated the searches using [valid*] instead of [validat*]. We applied filters for ‘past 10 years’ and ‘humans’. We excluded studies if they reported on an indication that was different from that for which authorisation was granted. We supplemented the PubMed searches with additional internet searches using Google and Google Scholar. Two investigators independently reviewed the search results. For discrepancies, we consulted a third investigator to reach consensus.

We applied F&P and Ciani hierarchies to categorise surrogate endpoints reported in the pivotal trials (Table 2) [7,8]. We carried out the first stage of the Ciani methods and omitted the steps that seek to establish a correlation coefficient for the surrogate–endpoint relationship. We did not include this step because our interest in the current study was to establish whether a surrogate endpoint had been validated or not as predictive of the clinical outcome, not to conduct a validation process. Moreover, correlations between surrogate endpoints and clinical outcomes may not reliably predict clinical efficacy [8,10–12,23]. Three authors categorised the pivotal trial endpoints independently according to both F&P and Ciani hierarchies and based on evidence from the literature searches. Where individual categorisations differed, we discussed to reach consensus. Surrogates of F&P ‘Level 2’ and Ciani ‘Level 1’ were considered validated. Where clinical outcomes were reported, these were categorised as Level 1 according to the F&P hierarchy. The Ciani hierarchy applies to surrogate endpoints only.

**Table 2:** Hierarchy of evidence for surrogate endpoint validity.

F&P (2012) [7]			Ciani (2017) [8]		
Level	Efficacy Measure	Evidence Requirement	Level	Evidence Requirement	Evidence Source
**1**	A true clinical efficacy	“When evidence establishing risk is acceptable in the context of evidence of benefit”	The Ciani hierarchy applies to surrogate endpoints only		
**2**	A validated surrogate (for a specific disease setting and class of interventions)	“When interventions are safe, with strong evidence that risks from off target effects are acceptable”	1	Treatment effect on surrogate corresponds to treatment effect on final endpoint	Randomised controlled trials showing that changes in the surrogate are associated with commensurate changes in the final endpoint
**3**	A nonvalidated surrogate, yet one established to be ‘reasonably likely to predict clinical benefit’ (for a specific disease setting and class of interventions)	“When interventions are safe, with evidence that risks from off target effects are acceptable”	2	Consistent association between surrogate and final endpoint	Epidemiological/observational studies
**4**	A correlate that is a measure of biological activity, but not established to be at a higher level	Not described	3	Biological plausibility of relation between surrogate and final endpoint	Pathophysiological studies and understanding of the disease process

**Abbreviations:** ‘Ciani’, Ciani and colleagues; F&P, Fleming and Powers

We further categorised pivotal trial endpoints according to whether they were single, composite, or multiple. Single outcomes were those for which the pivotal trial reported a single clinical or surrogate endpoint. A composite endpoint was that for which the pivotal trial reported an endpoint comprising more than one element and for which the individual elements could not be distinguished, for example, PFS, defined as the time to disease progression or death, whichever occurred first; because the numbers of deaths were not reported separately, the clinical component (death) could not be distinguished from the surrogate component, disease progression. Multiple endpoints arose when there was more than one pivotal trial supporting authorisation and these reported different primary endpoints or when a single trial reported more than one primary endpoint; for each endpoint, we recorded whether it was clinical or surrogate.

This study did not require ethics approval.

## Results

Between January 2011 and December 2018, 26 products granted CMA and 25 granted AA met the inclusion criteria (Table 3). Among the CMA products, 18 were to treat malignancy, 3 were for genetic, and 2 were for infection indications, and one each was for neurological, gastrointestinal, and endocrine disorders (Table 4). Among the products granted AA, 8 were to treat malignancy, 8 were for infection, 5 were for genetic disorders, 2 were for respiratory conditions, and 1 each was for a gastrointestinal disorder and a lymphoproliferative disorder (Table 4). There were 33 pivotal trials for CMA and 58 for AA products. Among the 51 products, 5 had pivotal trials that reported clinical outcomes, and 49 had trials with surrogate endpoints. The pivotal trial endpoints are described in detail in Table 4. No studies were identified that confirmed the validity of the surrogate endpoints as predicting the intended clinical outcomes (F&P Level 2 or Ciani Level 1).

**Table 3:** Numbers of products granted CMA and AA based on clinical and surrogate endpoints.

	CMA	AA	Total
**Number of product authorisations**	**26**	**25**	**51**
**Clinical endpoints (% authorisations)**	**0 (0%)**	**5 (20%)**	**5 (10%)**
Single endpoint: clinical	0	2	2
Multiple endpoints*			
Clinical plus nonvalidated surrogate	0	3	3
**Surrogate endpoints (% authorisations)**	**26 (100%)**	**20 (77%)**	**46 (90%)**
Validated endpoint	0	0	0
Nonvalidated endpoint			
Single endpoint	22	14	36
Composite endpoint**	4	6	10
**Total authorisations based on nonvalidated surrogate endpoints (% authorisations)**	**26 (100%)**	**20 (80%)**	**46 (90%)**

*Multiple endpoints whereby there was ≥1 pivotal trial and different primary endpoints were reported.

**Composite surrogate endpoint, e.g., PFS, defined as the time to death or progression of disease, but the numbers of deaths were not reported.

**Abbreviations:** AA, accelerated assessment; CMA, conditional marketing authorisation; PFS, progression-free survival.

**Table 4:** Summary characteristics of products granted CMA or undergoing AA (January 1, 2011–December 31, 2018), date of authorisation and authorisation pathway, active substance, indication, pivotal (main) study primary endpoint as described in the EPAR, whether studies reported a surrogate endpoint, number and phase of pivotal trials, number of Identified studies confirming validity of the surrogate endpoint (F&P Level 2 or Ciani Level 1), and surrogate endpoint categorisation according to F&P and Ciani hierarchies.

Date authorised (authorisation pathway)	Active substance (product) (EPAR document identifier)	Indication as described in the product EPAR	Primary endpoint of pivotal (main) trial/s as described in the product EPAR	Surrogate endpoint (Y, N, composite^#^, multiple)	Number and pivotal trial phase	Identified studies confirming validity of the surrogate endpoint	Surrogate category:F&P hierarchy Level 1–4	Surrogate category:Ciani Level 1–3
19/07/2011 (CMA) (Full MA granted 22/05/2017)	**fampridine** (Fampyra) (EMA/555661/2011) [57]	“[F]or the improvement of walking in adult patients with multiple sclerosis with walking disability (EDSS 4–7)” (p. 88)	“[T]he proportion of ‘consistent’ responders defined as patients with higher walking speed for at least three out of four visits during the double-blind period as compared to the maximum value among the non-treatment visits. Walking speed was based on the T25FW [Timed 25 Foot Walk] Test, wherein a patient was asked to walk as quickly as possible, safely, from one end to the other end of a clearly marked, unobstructed, 25-foot course. After a maximum rest of 5 minutes the test was repeated again. The walking speed for a particular study visit was the average of the walking speeds of the two trials performed. If one of the 2 trials could not be fulfilled then the walking speed for that visit was to be the walking speed from the completed trial.” (p. 34)	Y	2× Phase III	0	3	3
01/09/2011 (CMA) (Full MA granted 16/11/2015)	**everolimus** (Votubia) (EMA/646111/2011) [58]	“[T]reatment of patients aged 3 years and older with subependymal giant cell astrocytoma (SEGA) associated with tuberous sclerosis complex (TSC) who require therapeutic intervention but are not amenable to surgery. The evidence is based on analysis of change in SEGA volume. Further clinical benefit, such as improvement in disease-related symptoms, has not been demonstrated.” (p. 44, p. 103)	“[T]he change from baseline in volume of the primary SEGA lesion at 6 months after the start of treatment (or at the last available assessment if a patient ended treatment prior to this timepoint) as determined by central radiology review.” (p. 55)	Y	Phase II	0	4	3
16/02/2012 (CMA)	**vandetanib** (Caprelsa) (EMA/128072/2012) [59]	“[T]reatment of aggressive and symptomatic medullary thyroid cancer (MTC) in patients with unresectable locally advanced or metastatic disease. For patients in whom Rearranged during Transfection (RET) mutation is not known or is negative, a possible lower benefit should be taken into account before individual treatment decision (see important information in sections 4.4 and 5.1).” (p. 2)	“Progression-free survival (PFS), defined from the date of randomization to the date of objective progression or death (by any cause in the absence of progression), provided death was within 3 months from the last evaluable RECIST assessment, using data from RECIST assessments performed at baseline, during treatment and during the follow-up period. The PFS assessment was based on an independent radiological review.” (p. 35)	Y Composite: disease progression/death	Phase III	0	3	3
09/05/2012 (CMA)	**pixantrone dimaleate** (Pixuvri) (EMA/309145/2012) [60]	“[M]onotherapy for the treatment of adult patients with multiply relapsed or refractory aggressive Non Hodgkin B cell Lymphomas (NHL). The benefit of pixantrone treatment has not been established in patients when used as fifth line or greater chemotherapy in patients who are refractory to last therapy.” (p. 2)	“CR [complete response] or CRu [complete response unconfirmed] rate (ITT) [intention to treat] assessed by the Independent Assessment Panel (IAP) based on the Report of the International Workshop to Standardize Response Criteria. These criteria are also known as the International Working Group (IWG) criteria (Cheson 1999).” (p. 43)	Y	Phase III	0	4	3
22/10/2012 (CMA) (Full MA granted 15/09/ 2016)	**crizotinib** (Xalkori) (EMA/327604/2012) [61]	“[T]reatment of adults with previously treated anaplastic lymphoma kinase (ALK)-positive advanced non-small cell lung cancer (NSCLC).” (p. 2)	“ORR [objective response rate]…defined as the percent of patients in the Response Evaluable (RE) population achieving a confirmed CR [complete response] or confirmed PR [partial response] according to RECIST.” (p. 42)	Y	Phase I	0	4	3
24/10/2012 (CMA)	**brentuximab vedotin** (Adcetris) (EMA/702390/2012) [62]	“[T]reatment of adult patients with relapsed or refractory CD30+ Hodgkin lymphoma (HL): 1. following autologous stem cell transplant (ASCT) or 2. following at least two prior therapies when ASCT or multi-agent chemotherpay are not a treatment option ADCETRIS is indicated for the treatment of adult patients with relapsed or refractory systemic anaplastic large cell lymphoma (sALCL).” (p. 2)	SGO35-0003 “[T]he overall objective response rate (ORR) per an independent review facility (IRF). Treatment response was assessed by spiral CT of the chest, neck, abdomen, and pelvis and PET scans. Determination of antitumour efficacy was based on objective response assessments made according to the Revised Response Criteria for Malignant Lymphoma (Cheson et al, 2007), which includes radiographic disease assessment by computed tomography (CT) and/or positron emission tomography (PET) scans and oncology review of clinical data” (p.48) SGO35-0004 “[T]he overall ORR per IRF” (Similar methods as SGO35-0003) (p. 56)	Y	2× Phase II	0	4	3
26/03/2013 (CMA)	**bosutinib** as monohydrate (Bosulif) (EMA/70929/2013) [63]	“[T]reatment of adult patients with chronic phase (CP), accelerated phase (AP), and blast phase (BP) Philadelphia chromosome positive chronic myelogenous leukaemia (Ph+ CML) previously treated with one or more tyrosine kinase inhibitor(s) and for whom imatinib, nilotinib and dasatinib are not considered appropriate treatment options.” (p. 2)	“Clinical evaluation of efficacy CP [chronic phase] CML patients who were resistant to imatinib: • MCyR (PCyR or CCyR) at 24 weeks “(p. 46) (Major cytogenic response [partial cytogenic response or complete cytogenic response])	Y	Phase III	0	4	3
11/07/2013 (CMA) (Full MA granted 15/09/2016)	**vismodegib** (Erivedge) (EMA/297688/2013) [64]	“[T]reatment of adult patients with: - Symptomatic metastatic basal cell carcinoma - Locally advanced basal cell carcinoma inappropriate for surgery or radiotherapy” (p. 2)	“ORR [objective response rate] as assessed by independent review facility (IRF)” (p. 45)	Y	Phase II	0	4	3
04/03/2014 (CMA)	**bedaquiline fumarate** (Sirturo) (EMA/329898/2014) [65]	“[F]or use as an appropriate combination regimen for pulmonary multidrug resistant (MDR TB) in adult patients when an effective treatment regimen cannot otherwise be composed for reasons of resistance or tolerability” (p. 2)	“[T]ime to sputum culture conversion (SCC) during treatment with bedaquiline or placebo. This parameter was based on the qualitative assessment of culture growth in mycobacteria growth indicator tube using spot sputum samples” (p. 49)	Y	Phase II	0	3	3
20/03/2014 (CMA)	**cabozantinib** (Cometriq) (EMA/97103/2014) [66]	“[T]reatment of adult patients with progressive, unresectable locally advanced or metastatic medullary thyroid carcinoma” (p. 105)	“Progression-free survival (IRC [Independent Review Committee] determined) defined as the time from randomization to documented PD per m[odified] RECIST criteria or death due to any cause, whichever occurred first” (p. 46)	Y Composite: disease progression/death	Phase III	0	3	3
27/04/2014 (CMA)	**delamanid** (Deltyba) (EMA/55567/2014) [67]	“[F]or use as part of an appropriate combination regimen for pulmonary multi-drug resistant tuberculosis (MDR-TB) in adult patients when an effective treatment regimen cannot otherwise be composed for reasons of resistance or tolerability” (p. 136)	“[T]he proportion of the subset of MITT [modified intention to treat] subjects (sputum culture positive for MDR-TB at baseline) that achieved SCC [sputum culture conversion] using the MGIT [mycobacteria growth indicator tube] by Day 57. The time to SCC was based on the collection of the first sputum specimen with MGIT culture negative for growth of MTB that was followed by at least one additional sputum specimen with no MTB growth in MGIT at least 27 days after the first negative specimen and not followed by any sputum specimens with MGIT growth of MTB at any point during the remainder of the 84 day study period” (p. 52)	Y	Phase II	0	3	3
30/07/2014 (CMA)	**ataluren** (Translarna) (EMA/369266/2014) [68]	“[T]reatment of Duchenne muscular dystrophy resulting from a nonsense mutation in the dystrophin gene, in ambulatory patients aged 5 years and older” (p. 100)	“[C]hange in 6MWD [6-minute walk distance] from baseline to Week 48” (p. 32)	Y	Phase IIb	0	4	3
05/05/2015 (CMA) (Full MA granted 26/07/2017)	**ceritinib** (Zykadia) (EMA/170114/2015) [69]	“[T]reatment of adult patients with anaplastic lymphoma kinase-positive advanced non-small cell lung cancer previously treated with crizotinib” (p. 2)	“Overall response rate (ORR, CR+PR [complete response + partial response]) and duration of response (DOR) as assessed by the investigator per RECIST [Response Evaluation Criteria in Solid Tumours] 1.0” (p. 56)	Y	Phase I	0	4	3
22/11/2015 (CMA) (Full MA granted 18/06/2018)	**blina-tumomab** (Blincyto) (EMA/CHMP/469312/2015) [70]	“[T]reatment of adults with Philadelphia chromosome negative relapsed or refractory B-precursor Acute Lymphoblastic Leukaemia (ALL)” (p. 119)	“[T]he CR/CRh* [Complete remission/CR with partial hematological recovery] rate calculated as the number of subjects with either a CR or CRh* response within the first two treatment cycles divided by the total number of subjects in the analysis set” (p. 49)	Y	Phase II	0	4	3
01/02/2016 (CMA) (Full MA granted 28/04/2017)	**osimertinib mesylate** (Tagrisso) (EMA/CHMP/15445/20165) [71]	“[T]reatment of adult patients with locally advanced or metastatic epidermal growth factor receptor (EGFR) T790M mutation-positive non-small-cell lung cancer (NSCLC)” (p. 132)	Both studies: “ORR [objective response rate] according to RECIST 1.1 by BICR [blind independent central review] using the evaluable for response analysis set” (p. 64)	Y	2× Phase I/II	0	4	3
19/05/2016 CMA (Full MA granted 24/04/2017)	**daratumumab** (Darzalex) (EMA/278085/2016) [72]	“[T]reatment of adult patients with relapsed and refractory multiple myeloma, whose prior therapy included a proteasome inhibitor and an immunomodulatory agent and who have demonstrated disease progression on the last therapy2 (p. 117)	MMY2002: “[T]he overall response rate (ORR), which was defined as the proportion of subjects who achieved PR or better according to the IMWG [International Myeloma Working Group] criteria” (p. 47) GEN501: “[T]he overall response rate (ORR), which was defined as the proportion of subjects who achieved PR or better. Objective response evaluations were made based on assessments from a computerized algorithm using the International Multiple Myeloma Working Group (IMWG) Response Criteria for Multiple Myeloma” (p. 68)	Y	2× Phase II	0	3	3
17/08/2016 (CMA)	**Allogeneic T cells genetically modified with a retroviral vector** (Zalmoxis) (EMA/CHMP/589978/2016) [73]	“[A]djunctive treatment in haploidentical haematopoietic stem cell transplantation of adult patients with high-risk haematological malignancies” (p. 96)	“[T]he proportion of patients who achieved immune reconstitution, empirically defined a priori as an absolute CD3+ cell count of 100/μL or more for two consecutive observations (and/or CD4+ cells ≥ 50/μL and/or CD8+ cells ≥ 50/μL)” (p. 38)	Y	Phase I/II	0	4	3
09/11/2016 (CMA)	**olaratumab** (Lartruvo) (EMA/CHMP/742133/2016) [74]	“[I]n combination with doxorubicin for the treatment of adult patients with avanced soft tissue sarcoma who are not amenable to curative treatment with surgery or radiotherapy and who have not been previously treated with doxorubicin” (p. 113)	“PFS [Progression Free Survival] in patients with advanced STS [soft tissue sarcoma] not amenable to treatment with surgery or radiotherapy when treated olaratumab in combination with doxorubicin versus doxorubicin alone” (p. 55) PFS: time from the date of randomisation to the earliest date of documented tumour progression or death from any cause, whichever was first. Tumour assessment based on Response Evaluation Criteria in Solid Tumours 1.1 criteria as per Investigator	Y Composite: disease progression/death	Phase Ib/II	0	3	3
20/11/2016 (CMA)	**ixazomib citrate** **(**Ninlaro) (EMA/CHMP/594718/2016) [75]	‘‘[I}n combination with lenalidomide and dexamethasone” “for the treatment of adult patients with multiple myeloma who have received at least one prior therapy” (p. 147)	“Progression Free Survival defined as the time from the date of randomisation to the date of first documentation of disease progression, based on central laboratory results and International Myeloma Working Group criteria, or death due to any cause, whichever occurred first” (p. 73)	Y Composite: disease progression/death	Phase III	0	3	3
04/12/2016 (CMA) (Full MA granted 20/11/2018)	**venetoclax** (Venclyxto) (EMA/725631/2016) [76]	“[T]reatment of chronic lymphocytic leukaemia (CLL) in the presence of 17p deletion or TP53 mutation in adult patients who are unsuitable for or have failed a B-cell receptor pathway inhibitor” and “[T]reatment of CLL in the absence of 17p deletion or TP53 mutation in adult patients who have failed both chemoimmunotherapy and a B-cell receptor pathway inhibitor” (p. 131)	“ORR [overall response rate], the proportion of subjects with an overall response (CR + CRi + nPR + PR) [complete remission + complete remission with incomplete bone marrow recovery + nodular partial remission+ partial remission] per the NCI-WG [National Cancer Institute Working Group] guidelines as assessed by the IRC [Independent Review Committee] in the first 70 subjects enrolled treated in the main cohort” (p. 62)	Y	Phase II	0	4	3
11/12/2016 (CMA)	**obeticholic acid** (Ocaliva) (EMA/725757/2016) [77]	“[T]reatment of primary biliary cholangitis (also known as primary biliary cirrhosis) in combination with ursodeoxycholic acid (UDCA) in adults with an inadequate response to UDCA or as monotherapy in adults unable to tolerate UDCA” (p. 2)	“[T]he percentage of subjects (OCA [obeticholic acid] 10 mg vs. placebo) reaching an ALP [alkaline phosphatase] <1.67 x ULN [upper limit(s) of normal] and a ≥15% reduction in ALP and a total bilirubin ≤ULN at Month 12” (p. 56)	Y	Phase III	0	4	3
16/02/2017 (CMA)	**alectinib** (Alecensa) (EMA/197343/2017) [78]	“[M]onotherapy for adult patients with anaplastic lymphoma kinase (ALK) positive advanced non-small cell lung cancer (NSCLC) previously treated with crizotinib” (p. 121)	NP28761: “ORR [objective response rate] (IRC [Independent Review Committee] assessed)—Proportion of patients achieving confirmed CR [complete response] or PR [partial response]” (p. 61) NP28673: “ORR as per central IRC using RECIST v1.1 in the overall population (with and without prior exposure of cytotoxic chemotherapy treatments)” and “….in patients with prior exposure of cytotoxic chemotherapy treatments” (p. 73)	Y	2× Phase I/II	0	4	3
23/04/2017 (CMA)	**parathyroid hormone** (Natpar) (EMA/180882/2017) [79]	“[A]djunctive treatment of adult patients with chronic hypoparathyroidism who cannot be adequately controlled with standard therapy alone” (p. 108)	“[T]he percentage of subjects who met the triple efficacy endpoint at Week 24, based on investigator-prescribed data. A subject met the triple efficacy endpoint if he/she achieved:—At least a 50% reduction from the baseline oral calcium supplementation dose; and—At least a 50% reduction from the baseline active vitamin D metabolite/analog dose and—An albumin-corrected total serum calcium concentration that was maintained or normalized compared to the baseline value (≥ 1.875 mmol/L) and did not exceed the upper limit of the laboratory normal range” (p. 41)	Y	Phase III	0	4	3
17/09/2017 (CMA)	**avelumab** (Bavencio) (EMA/496593/2017) [80]	“[M]onotherapy for the treatment of adult patients with metastatic Merkel cell carcinoma” (p. 125)	“ORR [objective response rate] according to RECIST [Revised Evaluation Criteria in Solid Tumours] 1.1 as determined by an IIERC [Independent Endpoint Review Committee]” (p. 49)	Y	Phase II	0	4	3
19/02/2018 (CMA)	**burosumab** (Crysvita) (EMA/148319/2018) [81]	“Treatment of X-linked hypophosphataemia with radiographic evidence of bone disease in children 1 year of age and older and adolescents with growing skeletons” (p. 128)	Children: “Change from Baseline in severity of rickets as measured by Rickets Severity Score (RSS) total score” (p. 44) (RSS: Sum of scores assessed in hand/wrist and knee radiographs) (UX023-CL201 assessed at 64 weeks; UX023-CL205 at 40 weeks) Adults: “[T]he proportion of subjects achieving mean serum phosphorus levels above the lower limit of normal (0.81 mmol/L) at the mid-point of the dose interval (i.e. Weeks 2, 6, 10, 14, 18 and 22), as averaged across dose cycles between baseline and Week 24” (p. 82)	Y	2× Phase II	0	3	3
23/05/2018 (CMA)	**rucaparib camsylate** (Rubraca) (EMA/CHMP/238139/2018) [82]	“[M]onotherapy treatment of adult patients with platinum sensitive, relapsed or progressive, BRCA mutated (germline and/or somatic), high-grade epithelial ovarian, fallopian tube, or primary peritoneal cancer, who have been treated with two or more prior lines of platinum based chemotherapy, and who are unable to tolerate further platinum based chemotherapy” (p. 164)	CO-338-010 Part 2A: “ORR [objective response rate] defined as best confirmed response according to RECIST Version 1.1” CO-338-017 Part 1: “PFS according to RECIST Version 1.1, as assessed by the investigator, or death from any cause, in molecularly defined HRD subgroups” Part 2: “ORR by RECIST Version 1.1 in molecularly defined HRD subgroups” (p. 74)	Y	2× Phase I/II	0	4	3
04/09/2011 (AA)	**abiraterone acetate** (Zytiga) (EMA/CHMP/542871/2011) [83]	“[I]n combination prednisone or prednisolone in the treatment of metastatic castration resistant prostate cancer in adult men whose disease has progressed on or after a docetaxel-based chemotherapy regimen” (p. 77)	“Overall Survival (OS) defined as the interval from the date of randomization to the date of death from any cause. Survival follow-up was to continue every 3 months for up to 60 months (5 years) after the patient’s entry into the study” (p. 39)	N (survival)	Phase III	NA	1	NA
16/02/2012 (AA)	**vemurafenib** (Zelboraf) (EMA/200986/2012) [84]	“[M]onotherapy for the treatment of adult patients with BRAF-V600-mutation-positive unresectable or metastatic melanoma” (p. 2)	Two co-primary endpoints: “OS [Overall Survival] and PFS [Progression Free Survival]” (p. 47) PFS: the time from randomisation to the date of disease progression (based on tumour assessment date) or death from any cause, whichever occurred first.	Multiple: N (survival)	Phase III	NA	1	NA
				Y Composite disease progression/death		0	3	3
22/07/2012 (AA)	**ivacaftor** (Kalydeco) (EMA/473279/2012) [85]	“[T]reatment of cystic fibrosis in patients age 6 years and older who have a G551D mutation in the CFTR [cystic fibrosis transmembrane conductance regulator] gene” (p. 86)	In both pivotal studies: “[T]he absolute change from baseline in percent predicted FEV1 [forced expiratory volume in one second] through 24 weeks of treatment” (p. 40)	Y	2× Phase III	0	3	2
30/06/2013 (AA)	**ponatinib** (Iclusig) (EMA/220290/2013) [86]	“[[I]n adult patients with • chronic phase, accelerated phase, or blast phase chronic myeloid leukaemia (CML) who are resistant to dasatinib or nilotinib; who are intolerant to dasatinib or nilotinib and for whom subsequent treatment with imatinib is not clinically appropriate; or who have the T315I mutation • Philadelphia chromosome positive acute lymphoblastic leukaemia (Ph+ ALL) who are resistant to dasatinib; who are intolerant to dasatinib and for whom subsequent treatment with imatinib is not clinically appropriate; or who have the T315I mutation” (p. 2)	“For CML patients in CP at study entry: MCyR [major cytogenic response] defined as CCyR [complete cytogenic response] or PCyR [partial cytogenic response]; For CML patients in AP [accelerated phase] at study entry: MaHR [major haematologic response] defined as CHR [complete haematologic response] or NEL [no evidence of leukaemia]; For CML patients in BP [blast phase] at study entry or Ph+ ALL patients: MaHR consisting of CHR or NEL” (p. 42)	Y	Phase II	0	3	3
15/01/2014 (AA)	**sofosbuvir** (Sovaldi) (EMA/CHMP/688774/2013) [87]	“[I]n combination with other medicinal products for the treatment of chronic hepatitis C in adults” (p. 100)	“SVR12” (p66/p68/p69/p70) defined as “absence of measurable virus 12 weeks post end of treatment (SVR12)” (p. 9)	Y	4× Phase III	0	3	3
21/05/2014 (AA)	**siltuximab** (Sylvant) (EMA/CHMP/258608/2014) [88]	“[T]reatment of adult patients with multicentric Castleman’s disease (MCD) who are human immunodeficiency virus (HIV) negative and human herpesvirus-8 negative” (p. 2)	“[D]urable tumour and symptomatic response defined as either complete response (CR) or partial response (PR) as follows: PR was defined as a >50% decrease in sum of the product of the diameters (SPD) of index lesion(s), with at least [stable disease] SD in all other evaluable disease in the absence of treatment failure, sustained for at least 18 weeks. CR was defined as complete disappearance of all measurable and evaluable disease (e.g., pleural effusion) and resolution of baseline symptoms attributed to MCD, sustained for at least 18 weeks.” (p. 41)	Y	Phase II	0	4	3
22/08/2014 (AA)	**daclatasvir dihydro-chloride** (Daklinza) (EMA/419836/2014) [89]	“[I]n combination with other medicinal products for the treatment of chronic hepatitis C virus infection in adults” (p. 92)	“[T]he rate of sustained virologic response at follow-up Week 12 (SVR12) in each treatment group, where SVR12 was defined as HCV RNA less than the lower limit of quantitation, […] target detected […] or target not detected […] or at follow-up Week 12.” (p. 56)	Y	Phase II	0	3	3
16/11/2014 (AA)	**sofosbuvir/ledipasvir** (Harvoni) (EMA/702742/2014) [90]	“[T]reatment of chronic hepatitis C in adults” (79)	“[T]he antiviral activity of combination treatment with [sofosbuvir/ledipasvir] SOF/LDV with and without [ribavirin] RBV as measured by the proportion of subjects with sustained virologic response 12 weeks after discontinuation of therapy (SVR12), which in practice is equivalent to cure.” (p. 47)	Y	3× Phase III	0	3	3
14/01/2015 (AA)	**Ombitasvir** **/ paritaprevir/ ritonavir** (Viekirax) EMA/768346/2015) [91]	“[I]n combination with other medicinal products for the treatment of chronic hepatitis C in adults” (p. 149)	Five of the six pivotal trials assessed SVR12 in noncirrhotic patients with HCV genotype 1a and 1b infection; one trial assessed SVR12 in patients with compensated cirrhosis (p. 98)	Y	6 x Phase III	0	3	3
14/01/2015 (AA)	**nintedanib** (Ofev) (EMA/76777/2015) [92]	“[I]n adults for the treatment of Idiopathic Pulmonary Fibrosis (IPF)” (p. 84)	“[T]he annual rate of decline in FVC [forced vital capacity] (expressed in mL over 52 weeks)” (p. 59)	Y	2× Phase III	0	3	3
14/01/2015 (AA)	**dasabuvir sodium** (Exviera) (EMA/768319.2015) [93]	“[I]n combination with other medicinal products for the treatment of chronic hepatitis C in adults” (p. 119)	Study endpoints not stated in ‘Main Studies’ (Section 2.6.1) but were evident from ‘Discussion and Conclusions on clinical efficacy’ (p. 81–84): Sustained virologic response at follow-up week 12 (SVR 12).	Y	6× Phase III	0	3	3
27/05/2015 (AA)	**envatinib mesylate** (Lenvima) (EMA/250082/2015) [94]	“[T]reatment of adult patients with progressive, locally advanced or metastatic, differentiated (papillary/follicular/Hürthle cell) thyroid carcinoma, refractory to radioactive iodine (RAI)” (p. 2)	“PFS [progression-free survival], defined as the time from the date of randomisation to the date of first documentation of disease progression or death (whichever occurred first) as determined by blinded Independent Imaging Review (IIR) conducted by the imaging core laboratory using RECIST 1.1” (p. 82)	Y Composite disease progression/death	Phase III	0	3	3
27/08/2015 (AA)	**sebelipase alfa** (Kanuma) (EMA/514387/2015) [95]	“[F]or long-term enzyme replacement therapy (ERT) in patients of all ages with lysosomal acid lipase (LAL) deficiency” (p. 85)	“Proportion of subjects in the Primary Efficacy Set (PES) surviving to 12 months of age” (p.36) “Proportion of subjects who achieved ALT [alanine aminotransferase] normalisation (i.e., ALT below the ageand gender-specific ULN [upper limit of normal] provided by the central laboratory performing the assay) at the last visit in the double-blind treatment period.” (p. 39)	Multiple: N (survival)	Phase II/III	NA	1	NA
				Y	Phase III	0	4	3
18/11/2015 (AA)	**carfilzomib** (Kyprolis) (EMA/670306/2015) [96]	“[T]reatment of adult patients with multiple myeloma who have received at least one prior therapy in combination with lenalidomide and dexamethasone” (p. 139)	“PFS [progression-free survival] assessed by IRC [Independent Review Committee], defined as the duration in months from the date of randomization to the date of confirmed progressive disease (PD) or death due to any cause, whichever was earlier, according to the International Myeloma Working Group—Uniform Response Criteria (IMWG-URC).” (p. 50)	Y Composite; disease progression/death	Phase III	0	3	3
10/05/2016 (AA)	**elotuzumab** (Empliciti) (EMA/129497/2016) [97]	“[I]n combination with lenalidomide and dexamethasone for the treatment of multiple myeloma in adult patients who have received at least one prior therapy” (p. 109)	“PFS [progression-free survival] defined as the time from randomization to the date of the first documented tumour progression or death due to any cause as determined by independent review committee (IRC) using EBMT [European Group for Blood and Bone Marrow Transplant] criteria. The co-primary endpoint was ORR [objective response rate] defined as the proportion of randomized subjects who have either partial response or complete response as determined by IRC using the EBMT criteria.” (p. 43) “PFS defined as PFS is the time from randomization to the date of the first documented tumour progression or death due to any cause.” (p. 62)	Y Composite; disease progression/death	1× Phase III 1× Phase II	0	3	3
05/07/2016 (AA)	**sofosbuvir/velpatasvir** (Epclusa) (EMA/399285/2016) [98]	“[T]reatment of chronic hepatitis C” (p. 111)	“SVR12 [sustained virologic response at 12 weeks following completion of all treatment], defined as HCV RNA < LLOQ 12 weeks after discontinuation of the study drug, in all randomized and treated subjects…” (p. 73)	Y	6× Phase III	0	3	3
24/08/2016 (AA)	**lenvatinib** (Kisplyx) (None listed) [99]	“[I}n combination with everolimus for the treatment of adult patients with advanced renal cell carcinoma (RCC) following one prior vascular endothelial growth factor (VEGF)-targeted therapy” (p. 161)	“PFS” [defined as] “The time from randomization to the date of the first documented tumour progression as determined by the investigator using [Revised Evaluation Criteria in Solid Tumours] RECIST 1.1 criteria, or death due to any cause.” (p. 100)	Y Composite; disease progression/death	Phase II	0	3	3
08/09/2016 (AA)	**cabozantinib** (Cabometyx) (EMA/664123/2016) [100]	“[T]reatment of advanced renal cell carcinoma (RCC) in adults following prior vascular endothelial growth factor (VEGF)-targeted therapy” (p. 2)	“[D]uration of [Progression Free Survival] PFS as assessed by the [Independent Review Committee] IRC per [Revised Evaluation Criteria in Solid Tumours] RECIST 1.1, and was defined as the time from randomization to the earlier of the following events: documented PD [disease progression] per RECIST 1.1 or death due to any cause.” (p. 51)	Y Composite; disease progression/death	Phase III	0	3	3
29/05/2017 (AA)	**nusinersen** (Spinraza) (EMA/289068/2017) [101]	“[T]reatment of 5q Spinal Muscular Atrophy” (p. 116)	Study CS3B: “Proportion of motor milestone responders (Section 2 of the Hammersmith Infant Neurological Examination)” [and] “Time to death or permanent ventilation (≥16 hours ventilation/day continuously for >21 days in the absence of an acute reversible event OR tracheostomy).” (p. 47–48) Study CS4: “[C]hange from baseline in Hammersmith Functional Motor Scale–Expanded (HFMSE) score at 15 months” (p. 70)	N Multiple CS3B: Clinical (Motor Milestones/Death/Ventilation)	2× Phase III	0	1	NA
				Y			3	3
25/07/2017 (AA)	**glecaprevir/pibrentasvir** (Maviret) (EMA/449689/2017) [102]	“Treatment of chronic hepatitis C virus (HCV) infection in adults” (p. 125)	“[S]ustained virologic response 12 weeks post dosing [SVR12]” (p. 69)—in all 6 trials	Y	6× Phase III	0	3	3
25/07/2017 (AA)	**sofosbuvir/velpatasvir/voxilaprevi** (Vosevi) (None listed) [103]	“Treatment of chronic hepatitis C virus (HCV) infection in adults” (p. 152)	SVR12—in all 4 trials (p. 80) (p. 86) (p. 88) (p. 101)	Y	4× Phase III	0	3	3
08/01/2018 (AA)	**budesonide** (Jorveza) (EMA/774645/2018) [104]	“[T]reatment of eosinophilic esophagitis (EoE) in adults (older than 18 years of age)” (p. 2)	“[T]he rate of patients with clinico-pathological remission at week 6 (LOCF) defined as fulfilling both of the following criteria: Histological remission, i.e., peak of <16 eos/mm^2^ hpf at week 6 (LOCF), AND resolution of symptoms (i.e., no or only minimal problems). In addition, any patient in need of endoscopic intervention (e.g. for food impaction or dilation) was counted as treatment failure).” (p. 43)	Y Composite (Histology & Symptom score)	Phase III	0	3	3
05/07/2018 (AA)	**inotersen sodium** (Tegsedi) (EMA/381704/2018) [105]	“Treatment of Stage 1 or Stage 2 polyneuropathy in adult patients with hereditary transthyretin amyloidosis (hATTR)” (p. 138)	“Changes from Baseline to Week 66 in modified Neuropathy Impairment Score +7 (mNIS+7) and in the Norfolk Quality of Life-Diabetic Neuropathy (Norfolk QoL-DN) questionnaire total score.” (p. 69)	Y	Phase II/III	0	3	2
27/08/2018 (AA)	**patisiran sodium** (Onpattro) (EMA/554262/2018) [106]	“[T]reatment of hereditary transthyretin-mediated amyloidosis (hATTR amyloidosis) in adult patients with stage 1 or stage 2 polyneuropathy” (p. 186)	“[C]hange from baseline of mNIS+7 score at 18 months. The mNIS+7 is a composite assessment that measures a range of motor, sensory, and autonomic neurologic impairment experienced by hATTR-PN [with polyneuropathy] patients.” (p. 75)	Y	Phase III	0	3	2
22/11/2018 (AA)	lanadelumab (Takhzyro) EMA/794314/2018) [107]	“[R]outine prevention of recurrent attacks of hereditary angioedema (HAE) in patients aged 12 years and older” (p. 113)	“Number of investigator-confirmed HAE attacks during the treatment period (Day 0 through Day 182).” (p. 44)	N (Clinical: HAE attacks)	Phase III	NA	1	NA

#Composite endpoint: for example, PFS, whereby the endpoint was a combination of death or progression of disease—these elements were not reported separately; multiple endpoints whereby the pivotal trial/s reported distinct separate endpoints that were reported individually.

“NA” indicates not applicable (Ciani hierarchy does not include clinical outcomes; no literature searches were conducted for clinical outcomes).

**Abbreviations:** AA, accelerated assessment; ‘Ciani’, Ciani and colleagues [8]; CMA, conditional marketing authorisation; F&P, Fleming and Powers [7]; FEV1, forced expiratory volume in 1 second; FVC, forced vital capacity; HAE, hereditary angioedema; HCV, hepatitis C virus; MA, marketing authorisation; N, no; NA, not applicable; PFS, progression-free survival; RECIST, Revised Evaluation Criteria in Solid Tumours; SVR12, sustained virological response at 12 weeks following the end of treatment; Y, yes.

### CMAs

No product was granted CMA on the basis of a trial that reported a clinical endpoint. The pivotal trials reported a single nonvalidated surrogate endpoint for 22 CMA products and a composite nonvalidated endpoint for 4.

Of the 18 oncology products, the CMAs for 14 were based on pivotal trials reporting single surrogate endpoints. These included ‘objective response rate’ (reported for crizotinib, Xalkori; brentuximab vedotin, Adcetris; vismodegib, Erivedge; osimertinib, Tagrisso; alectinib, Alecensa; avelumab, Bavencio; rucaparib, Rubraca), overall response rate (ceritinib, Zykadia; daratumumab, Darzalex; venetoclax, Venclyxto), ‘complete response’ (pixantrone dimaleate, Pixuvri), ‘complete remission’ (blinatumomab, Blincyto), and ‘major cytogenic response’ (bosutinib, Bosulif) (Table 4). For 4 oncology products, the pivotal trials reported the composite endpoint of PFS: vandetanib (Caprelsa) and cabozantinib (Cometriq) for thyroid medullary cancer, olaratumab (Lartruvo) for soft tissue sarcoma, and ixazomib (Ninlaro) for multiple myeloma (Table 4).

Both fampridine (Fampyra) for multiple sclerosis and ataluren (Translarna) for Duchenne muscular dystrophy were granted CMA based on timed walking tests. The pivotal trial endpoints for 2 products indicated for multidrug-resistant tuberculosis—bedaquiline (Sirturo) and delamanid (Deltyba)—were based on sputum culture tests. For everolimus (Votubia), indicated for tuberous sclerosis-associated angiomyolipoma, the change in astrocytoma volume was reported, and for burosomab (Crysvita) (indicated for X-linked hypophosphotaemia), the rickets severity score based on knee and hand/wrist radiographs was reported. The pivotal trials for both obeticholic acid (Ocaliva) for primary biliary cirrhosis and parathyroid hormone (Natpar) for hypoparathyroidism reported biomarker endpoints.

In the F&P hierarchy, the endpoints in the pivotal trials for all 26 CMA products were categorised as Level 3 (the surrogate is established to be reasonably likely to predict clinical benefit for a specific disease setting and class of intervention) or Level 4 (the correlate is established to be a measure of biological activity but not established to be at a higher level) [7] (Table 4). In the Ciani hierarchy, all of the endpoints were categorised as Level 3, meaning there was biological plausibility of a relation between the surrogate and the clinical outcome [8].

Eight products were supported by Phase III trials, 11 by Phase II trials, 5 by Phase I/ II trials and 2 by Phase I trials alone (Table 4). By December 2018, 9 products granted CMA during the study period had been switched to full authorisation (Table 4 and S1 Table).

Three CMA products were also granted an AA—osimertinib mesylate (Tagrisso) indicated for non-small cell lung cancer, daratumumab (Darzalex) for multiple myeloma, and olaratumab (Lartruvo) for sarcoma. Osimertinib mesylate and daratumumab were fully authorised by December 2018.

### AAs

The pivotal trials for 5 of the 25 products granted AA reported clinical endpoints. For 2 products, this was a single endpoint, overall survival—abiraterone (Zytiga), for prostate cancer, and lanadelumab (Takhzyro), for hereditary angioedema (Tables 3 and 4). For 3 products—vemurafenib (Zelboraf) for melanoma, sebelioase alfa (Kanuma) for lysosomal acid lipase deficiency, and nusinersen (Spinraza) for spinal muscular atrophy—multiple different endpoints were reported in more than one trial, and for all 3, these included clinical endpoints as well as nonvalidated surrogate endpoints (Tables 3 and 4).

For 20 products, authorisation via AA was granted on the basis of trials reporting single nonvalidated surrogate endpoints (Tables 3 and 4). Eight of the products were for treatment of chronic hepatitis C virus (HCV) infection, and the pivotal trials were based on a biomarker, sustained virological response at 12 weeks following the end of treatment (SVR12). Among 7 oncology products, 5 had pivotal supporting trials that reported the composite outcome of PFS. The endpoints in the pivotal trials for 2 respiratory products, nintedanib (Ofev) for idiopathic pulmonary fibrosis and ivacaftor (Kalydeco) for cystic fibrosis, were forced vital capacity (FVC) and forced expiratory volume, respectively. The trials for 2 products for hereditary transthyretin amyloidosis, inotersen (Tegsedi) and patisiran (Onpattro), reported changes from baseline in neuropathy impairment scores. The pivotal trial endpoint for budesonide (Jorveza), indicated for eosinophilic esophagitis, was a composite of histology and symptom scores (Table 4).

The pivotal trial endpoints were categorised as F&P Level 1 for the 5 AA products where the trials reported clinical outcomes (Table 4). (The Ciani hierarchy does not apply for clinical endpoints.) The endpoints of forced expiratory volume in 1 second (FEV1) change reported for ivacaftor (Kalydeco) for cystic fibrosis and the neuropathy impairment scores reported for inotersen (Tegsedi) and patisiran (Onpattro) for hereditary transthyretin amyloidosis were categorised as F&P Level 3 and Ciani Level 2. Among the remaining products, most endpoints were categorised as F&P Level 3 and as Ciani Level 3 (Table 4).

Twenty products were supported by Phase III trials, one by a Phase II/III trial and 4 by Phase II trials (Table 4).

### EPAR explanation of rationale for pivotal trial endpoints

In some cases, EPARs mentioned that the pivotal trial endpoints evaluated to support authorisation recommendations were not clinical outcomes but did not systematically indicate whether surrogate endpoints were validated or nonvalidated. S1 Table summarises the information provided in each product EPAR on the rationale for the pivotal trial endpoint(s). Among CMA products, 18 of the 26 EPARs (69%) made no mention of whether the surrogate endpoint was validated or not. Others acknowledged uncertainties or highlighted difficulties in endpoint choice. For example, for multidrug-resistant tuberculosis, in the case of bedaquiline (Sirturo), the ‘time to sputum culture conversion’ (SCC) endpoint was acknowledged as a surrogate, but there was no mention of its validity in predicting the desired clinical outcome, while for delamanid (Deltyba), its association with SCC at 24 months and with mortality were discussed (S1 Table). For some products, preference was expressed for a different outcome, e.g., pixantrone (Pixuvri) for non-Hodgkin lymphoma, where it was noted that PFS or overall survival would have been preferred over the ‘complete response’ endpoint reported.

Among products granted AA, the EPARs for 13 of the 20 products (65%) with pivotal trial surrogate endpoints had no mention of the endpoint validity (S1 Table). In some cases, the rationale for the reported endpoints was provided. For example, in the case of ivacaftor (Kalydeco) for cystic fibrosis, the endpoint ‘change in percent predicted FEV1’ was described as ‘accepted’, and FEV1 rate of decline was described as ‘demonstrated to correlate with survival and to be the strongest clinical predictor of mortality’, but no supporting reference was provided (S1 Table). For the 9 products indicated for treatment of chronic HCV infection, the endpoint in the pivotal trials was SVR12, and the EPARs noted variously that this was accepted or followed established principles but did not explain details or describe limitations. For example, with sofosbuvir/ledipasvir (Harvoni), it was noted that SVR12 ‘in practice is equivalent to cure’, but no explanation was provided to support this view.

In contrast, for budesonide (Jorveza), indicated for eosinophilic esophagitis, the rationale for the pivotal trial endpoint was clearly stated: ‘At the time of conduct of trial, no obviously “standardised” method to assess the course of the symptoms was available, and therefore the chosen parameters appear to be adequate’; a prior EMA Scientific Advice that had contributed to endpoint discussions and choice was acknowledged (S1 Table).

### Postauthorisation measures

Among CMAs, Section 4 of the EPARs (and Annex ll, Section E of the SpCs) summarised the specific obligations required to complete postauthorisation measures, but these did not consistently specify whether the measures were intended to confirm clinical outcomes (S1 Table). For 4 of the 26 CMA products (15%), the required postauthorisation studies had a stated clinical endpoint, crizotinib (Xalkori) for non-small cell lung cancer, brentuximab vedotin (Adcetris) for myeloid leukaemia, ixazomib citrate (Ninlaro) for multiple myeloma, and obeticholic acid (Ocaliva) for primary biliary cirrhosis.

For 2 products, everolimus (Votubia) for tuberose sclerosis and vandetanib (Caprelsa) for thyroid neoplasm, the required postauthorisation studies had nonvalidated surrogate endpoints.

The endpoint that was to be reported in the required postauthorisation studies was not stated in the EPAR Section 4 information for 19 products granted CMA (73%). For example, in the case of daratumumab (Darzalex) for multiple myeloma, the specific obligation was written thus: “In order to address the uncertainties related to the single arm design of the pivotal study supporting the approval of Darzalex, the MAH [marketing authorisation holder] should submit the results of study MMY3003, a phase III randomised study investigating lenalidomide and dexamethasone with or without daratumumab in patients with previously treated multiple myeloma.” There was a similarly worded obligation to also provide the results of study MMY3004.

For the 25 products granted AA, Section 4 of the EPARs (and Annex ll, Section D of the SpCs) likewise indicated whether there were obligations to conduct postauthorisation measures (S1 Table)—these were obligations additional to the periodic safety update report (PSUR) and the activities and interventions detailed in the RMP. Two of the 5 (40%) products whose pivotal trials reported clinical outcomes had obligations to conduct postauthorisation studies, nusinersen (Spinraza) for spinal muscular atrophy and sebelipase alfa (Kanuma) for lysosomal acid lipase deficiency (S1 Table).

The pivotal trials for 20 of the 25 AA products (80%) reported surrogate endpoints of which 10 (50%) had postauthorisation obligations. For the 8 direct-acting antiviral products indicated for chronic HCV infection, the obligation was to conduct a prospective PASS to evaluate the recurrence of hepatocellular carcinoma associated with use of the products, a clinical safety outcome, not a clinical efficacy/effectiveness outcome. Studies with clinical outcomes were required for 2 products: ivacaftor (Kalydeco) for cystic fibrosis and siltuximab (Sylvant) for Castleman disease. The remaining 10 (50%) products had no obligations beyond their PSUR and RMP requirements. In total, 18 (90%) of the 20 products granted AA based on pivotal trials reporting surrogate endpoints had no requirement to conduct postauthorisation measures to confirm clinical efficacy/effectiveness outcomes.

## Discussion

In this study, we found that most of the marketing authorisations issued between January 1, 2011, and December 31, 2018, for products assessed through 2 EMA expedited pathways, CMA and AA, were based on pivotal trials that reported nonvalidated surrogate endpoints. This information was not systematically or explicitly included in the product EPARs, including in the SpCs, the definitive, regulator-approved information for prescribers (SpC Annex 1) and patients (SpC Annex IIIB, Package Leaflet). Prescribers and patients may therefore not be aware that there is only limited evidence that the products concerned provide clinical benefit. Moreover, clinical benefit may never be established because in most cases, there was also no requirement for marketing authorisation holders to conduct confirmatory postauthorisation studies.

Other studies, mainly in the US, have highlighted the use of surrogate endpoints in supporting expedited authorisations of oncology products and the subsequent failure to confirm clinical outcomes [24–28]. This study demonstrates that a similar situation applies for non-oncology products as well as for oncology products authorised in Europe through expedited pathways.

For products granted CMA based on pivotal trials reporting surrogate endpoints, the EPARs and SpCs (including the prescriber and patient information sections) typically provided no indication of whether the surrogates were validated or not as being predictive of the intended clinical outcomes. While it was always clear that more evidence was pending and postauthorisation monitoring was being undertaken, EPARs did not consistently say whether the associated specific obligations were intended to confirm clinical benefits. The required postauthorisation studies were described in Section 4 of the EPARs, but their outcomes, in many, were not stated. Highlighting the necessity of ensuring that clinical benefits are achieved from products authorised using surrogate endpoints, olaratumab (Lartruvo), granted CMA for the treatment of soft tissue sarcoma on the basis of PFS, a nonvalidated surrogate endpoint, failed to show a survival benefit in the postauthorisation study imposed as a specific obligation [29]. (The EPAR did not state the outcome to be evaluated in this study.) In April 2019, EMA recommended withdrawal of the marketing authorisation for olaratumab, noting that this was first revocation of a CMA [30].

While it was unsurprising that none of the products granted CMA were authorised on the basis of clinical outcomes, it was astonishing that just 20% of the products granted AA were supported by pivotal trials reporting clinical outcomes. For products authorised via the AA pathway, postauthorisation requirements may relate only to safety update reporting and to the RMP, and this was the case for half of the AA products recommended for authorisation on the basis of pivotal trials reporting surrogate endpoints. The EPARs and SpCs did not explain that clinical benefits might never be confirmed. For AA products for which postauthorisation measures were required, it was clear that ‘additional monitoring’ was being undertaken, but the documents did not distinguish whether this was to confirm clinical efficacy/effectiveness or evaluate safety outcomes.

### Categorisation of surrogate endpoints and regulator views on endpoints

We assessed surrogate endpoint validity according to the hierarchies of F&P and Ciani [7,8]. We judged surrogates of F&P Level 2 or Ciani Level 1 as validated. We did not find evidence at these levels for the endpoints reported in the pivotal trials of the CMA and AA products examined. In most cases, there was biological plausibility of a relation between the surrogate and the intended clinical outcome or a ‘reasonably likely’ relationship (Level 3 in both hierarchies).

None of the surrogate endpoints had an EMA qualification opinion [22], but for several conditions, we found that EMA guidance on treatment endpoints existed though it was not consistently mentioned in the corresponding product EPARs. For instance, among direct-acting antiviral products for chronic HCV infection, the primary endpoint in the pivotal trials, SVR12, was a nonvalidated surrogate endpoint, F&P Level 3 and Ciani Level 3 according to our methods. EMA guidance (2016) recommended SVR12 as the primary efficacy endpoint ‘for studies aiming at defining cure rate’ but offered no evidence to support its validity for this purpose and appears to recognise the limitations of SVR in specifying that ‘a representative subset’ of patients achieving SVR12 should be monitored for 12 months from end of treatment to assess durability, while those not achieving SVR12 should be monitored for 3 years [31]. Whilst SVR is a widely used endpoint in chronic HCV treatment trials and observational data from a single US-based population describe an association with mortality outcomes, there is debate about whether it reflects long-term clinical outcomes [7, 32–35]. A comprehensive review of randomised trials of direct-acting antivirals for chronic HCV infection concluded that ‘SVR is still an outcome that needs proper validation in randomised clinical trials’ [32].

In the case of cystic fibrosis, the primary endpoint in the pivotal trial for ivacaftor (Kalydeco) was the rate of decline in FEV1, a surrogate endpoint associated with morbidity and mortality and categorised as F&P Level 3 and Ciani Level 2 according to our methods. Challenges in its use are well-acknowledged, and an EMA-hosted cystic fibrosis stakeholder workshop in 2012 reported that ‘FEV1, despite its major limitations, still remains an important outcome measure for clinical efficacy’ [36, 37]. The workshop was not mentioned in the EPAR or SpC.

### Surrogate categorisations: Evidence from the literature

The surrogate endpoint of SCC within 2 months, reported in the pivotal studies for 2 products granted CMA to treat multidrug-resistant tuberculosis, bedaquiline fumarate (Sirturo) and delamanid (Deltyba), has been shown to correlate well with recurrence of infection in drug-sensitive tuberculosis, but data on its reliability in multidrug-resistant tuberculosis were unavailable, hence we categorised it as F&P Level 3, Ciani Level 3 [38].

Nintedanib (Ofev), granted AA for treatment of idiopathic pulmonary fibrosis, was similarly categorised. The pivotal trials used the endpoint ‘annual rate of decline in FVC’. Discussions in the literature consider options for analysing FVC changes; although FVC of itself has been shown to predict mortality, its annual rate of decline has not been shown to predict disease progression [39, 40].

For the neuropathy impairment scores reported for inotersen (Tegsedi) and patisiran (Onpattro) for hereditary transthyretin amyloidosis, the Norfolk quality of life score demonstrated correlation with clinical outcomes in an observational study, while in a subset of patients recruited to a randomised trial, it was shown that clinicians could be trained to accurately assess neuropathy signs using a modified neuropathy impairment score (mNIS+7_Ionis_) [41, 42]. We categorised these endpoints as F&P Level 3 and Ciani Level 2.

Among the 17 CMA and 8 AA oncology products, most pivotal trials reported surrogate endpoints of F&P Level 4 and Ciani Level 3 according to our methods. Correlations between surrogate endpoints and clinical outcomes have been investigated quite extensively in oncology treatments, especially between the composite surrogate endpoint, PFS, and overall survival, and found wanting [10, 24, 25, 43, 44]. Heterogeneous correlations are reported across studies, even where the same endpoint was investigated in the same cancer type [10]. Using a validated framework—the European Society for Medical Oncology Magnitude of Clinical Benefit Scale (ESMO-MCBS)—to assess the ‘meaningful clinical benefit’ of 38 oncology treatments authorised for 70 indications between 2011 and 2016, the threshold for benefit was achieved in only 21% of the indications [45]. When a more rigorous but nonvalidated version of the framework was applied, just 11% met the threshold. The endpoints in the trials for 8 CMA products and 3 AA products were assessed according to the Revised Evaluation Criteria in Solid Tumours (RECIST), a set of rules for reporting of treatment responses for solid tumours [46]. RECIST aims to ensure consistency in measuring progression of disease but does not assess surrogate endpoint validity.

EMA guidance on the evaluation of oncology products states that there should be ‘sufficient evidence available demonstrating that the chosen primary endpoint can provide a valid and reliable measure of clinical benefit’ noting that ‘convincingly demonstrated favourable effects on survival’ were ‘the most persuasive outcome of a clinical trial’ [47]. ‘Acceptable’ primary endpoints in Phase III confirmatory trials were cure rate, overall survival, and PFS/disease-free survival, but we found no discussion on the validity of PFS in reflecting clinical outcomes. Objective response rate, the pivotal trial endpoint for 7 CMA products, was judged suitable in Phase II evaluation of activity studies, but its validity as a surrogate was not discussed. Neither overall response nor complete response/remission, the pivotal trial endpoints for 5 products, was mentioned [47].

Thus, where product authorisation was supported by pivotal trials reporting surrogate endpoints, the major issue of concern highlighted by our findings is that the EPARs, including the SpCs, did not clearly describe whether the surrogates were validated as reliably reflecting clinical outcomes. This is an omission that could feasibly be addressed in both documents. Our findings do not mean that the appropriateness of pivotal trial endpoints was not discussed during regulatory evaluations, but if it was discussed, the reasoning was not systematically recorded in the main public assessment report or in the SpC.

Surrogate endpoint validity is a matter of concern for decision-makers other than medicines regulators. For example, the National Institute for Health and Care Excellence (NICE) in the United Kingdom recommends that ‘when the use of “final” clinical endpoints is not possible and “surrogate” data on other outcomes are used to infer the effect of treatment on mortality and health-related quality of life, evidence in support of the surrogate-to-final endpoint outcome relationship must be provided together with an explanation of how the relationship is quantified’ [48]. EUnetHTA, the European network of health technology assessment agencies, has provided guidance on the use of surrogate endpoints in relative effectiveness assessment of pharmaceuticals [49]. In Germany, the Institute for Quality and Efficiency in Healthcare has published recommendations for the validation of surrogate endpoints in oncology, has documented the limited benefits of new medicines entering the German healthcare system, and called for EU and national action to define public health goals and to revise the legal and regulatory framework to properly meet patient needs [50, 51].

### Benefits and drawbacks of expedited authorisations

Studies investigating the additional therapeutic value of products authorised through expedited pathways have variable findings. A study of expedited drug development and approval programs in the US, 1987–2014, criticised the regulator’s oversight of applications to the programs after finding that increasing numbers were driven by drugs not first in class and therefore not likely to provide noticeable clinical advances over existing products [17]. Quality adjusted life year gains have been reported with some expedited authorisations compared with standard approvals in US healthcare settings, but safety problems leading to market withdrawal of products have also emerged [26, 27, 50]. Although a study of products authorised via 2 EMA expedited pathways compared with standard authorisations, 1999–2009, reported no increase in postmarketing safety alerts or withdrawal, in terms of clinical value, it appears that most new cancer drugs authorised on the basis of surrogate outcomes provided little or no survival or quality of life benefit [27, 28, 52, 53].

### Postauthorisation requirements in expedited authorisations

A recurring criticism of expedited authorisations is the failure by marketing authorisation holders to undertake and/or complete postmarketing obligations in a timely and rigorous manner, both in the EU and the US [5,14–18,27, 54–56]. Notably, completed postapproval studies of oncology products granted accelerated approval in the US (2009–2013) mainly reported surrogate endpoints, not clinical outcomes, highlighting that without ensuring that surrogates are validated, clinical benefit may never be established [24, 25, 27]. In our study, which examined products for oncology and beyond, the EPARs in most cases did not state the endpoint for the postmarketing studies imposed as specific obligations on products granted CMA. Of the 6 products for which the endpoints were stated, these were clinical outcomes for 4 products, but nonvalidated surrogate endpoints were required for 2 products.

Products granted AA may have no postmarketing requirements beyond PSUR and RMP activities. Of the 20 out of 25 AA products in our study that had pivotal trials reporting surrogate endpoints, 18 (90%) had no requirement to undertake postmarketing measures to confirm clinical efficacy/effectiveness (rather than safety) outcomes. Thus, their clinical benefits may never be known.

### Limitations

The main limitation of the study is that the findings apply to products recommended for authorisation through 2 expedited assessment pathways and may not be generalisable to products authorised through the standard pathway. For the CMA and AA products examined, the EPAR documentation is extensive, and it is possible we overlooked some information (or products) during data collection. We tried to minimise subjectivity in our decisions on whether surrogate endpoints were validated or nonvalidated by searching the literature widely for supporting validation studies, but we may not have found all of the relevant studies. We further sought to minimise subjectivity by applying methods from 2 independent systems to categorise surrogate endpoints. Where individual author categorisations differed, we reached agreement by consensus but acknowledge the final categorisation assumed that our searches had found all of the relevant studies of endpoints. We searched for EMA qualification opinions or other guidance relating to the endpoints reported in pivotal trials. We found guidance in 2 cases that helped to explain the endpoint choice although neither was mentioned in the associated product EPARs, but we may have overlooked other cases.

We focused on the primary endpoints reported in the pivotal supporting trials and did not collect information on secondary endpoints or on outcomes of nonpivotal trials. These may have reported some clinical outcomes but were obviously not adequate as primary evidence; otherwise, they would have been considered as such.

## Conclusion

This is, to our knowledge, the first systematic study of the use of surrogate endpoints to support marketing authorisation of products assessed through both CMA and AA expedited regulatory pathways in the EU. The extensive use of nonvalidated surrogate endpoints is concerning because the likelihood that treatment will provide the intended clinical benefit is unknown. In the current study, it was not clear from the publicly available information about the products whether surrogate endpoints were validated or nonvalidated. Neither was it clear whether postauthorisation measures—when they were required—would confirm clinical benefits. Because products authorised through these pathways are intended to satisfy unmet need or are in the public interest, the marketing authorisation holders should ultimately be required to demonstrate that they fulfil these goals.

It would be helpful for patients, prescribers, and healthcare providers broadly if the regulator ensured that EPARs, including SpC Annex I (Prescriber information) and SpC Annex III (Package leaflet), as well as the website ‘authorisation details’ summary, consistently provide explicit information on the nature of the pivotal study endpoints supporting marketing authorisations. When surrogate endpoints are used, this needs to indicate their level of validity, the rationale for their acceptance (including whether there was a qualification opinion, whether scientific advice had been provided, or whether EMA had otherwise provided guidance), and their limitations in reflecting intended clinical outcomes. If surrogate endpoints have supported the authorisation, then postauthorisation measures to be completed in a reasonable time frame are needed, including for rare disease treatments, to confirm that the clinical outcomes are achieved. When such measures fail to confirm clinical benefit, then—as in the case of olaratumab—the regulator should consider withdrawal of the marketing authorisation.

## Supporting information

S1 Text Study protocol. (DOCX)

S1 Table Summary information on products granted CMA or undergoing AA (January 1, 2011–December 31, 2018). AA, accelerated assessment; ‘Ciani’, Ciani and colleagues; CMA, conditional marketing authorisation; EPAR, European Public Assessment Report; F&P, Fleming and Powers; HCC, hepatocellular carcinoma; NA, not applicable; PASS, postauthorisation safety study; PSUR, periodic safety update report; RMP, risk management plan; List of excluded products.
(XLSX)

## Abbreviations

AA: accelerated assessment
‘Ciani’: Ciani and colleagues
CMA: conditional marketing authorisation
EMA: European Medicines Agency
EMSO-MCBS: European Society for Medical Oncology Magnitude of Clinical Benefit Scale
EPAR: European Public Assessment Report
EU: European Union
EUnetHTA: European network for Health Technology Assessment
FEV1: forced expiratory volume in 1 second
F&P: Fleming and Powers
FVC: forced vital capacity
HCV: hepatitis C virus
mNIS: modified neuropathy impairment score
NICE: National Institute for Health and Care Excellence
PASS: postauthorisation safety study
PFS: progression-free survival
PSUR: periodic safety update report
RECIST: Revised Evaluation Criteria in Solid Tumours
RMP: risk management plan
SCC: sputum culture conversion
SpC: summary of product characteristics
SVR12: sustained virological response at 12 weeks following the end of treatment
