# Supplementary material for: The use of validated and nonvalidated surrogate endpoints in two European Medicines Agency expedited approval pathways: A cross-sectional study of products authorised 2011–2018
Source: PLoS Med. 2019 Sep 10;16(9):e1002873. doi: 10.1371/journal.pmed.1002873 (PMC6736244; doi:10.1371/journal.pmed.1002873)
Supplement: S1 Text — (DOCX) [file pmed.1002873.s001.docx]

**The use of validated and non-validated surrogate endpoints in two European Medicines Agency expedited approval pathways**

**A cross-sectional study of products authorised 2011-2018**

**Study Protocol**

**Research question**

Are EMA recommendations for marketing approvals via two expedited pathways, conditional marketing approval (CMA) and accelerated approval (AA) based on pivotal trials reporting clinical outcomes or surrogate endpoints.

For products where surrogate endpoints are reported, is there evidence that in each case the endpoint is validated as reflecting the intended clinical outcome?

**Protocol**

- Identify products granted CMA or AA by EMA, January 2011 – December 2017 (extended to 2018) through EMA website and published reports
  - Exclusions: vaccines, reversal agents, prophylaxis agents, withdrawn products
- Search of individual product European Public Assessment Reports (EPARs) to identify for each product the approved indication, pivotal trial/s primary endpoint/s, specific obligations and / or other post-marketing requirements
- For products with pivotal trial/s reporting surrogate endpoint/s, PubMed searches for studies validating the endpoint
  - Search terms: [‘endpoint’] and [validat* surrogate outcome OR validat* surrogate endpoint OR validat* surrogate end-point] and [‘indication’] (i.e. the ‘therapeutic indication’ described in ‘product information’ provided in the EMA website).
  - Search filters: ‘past 10 years’ and ‘humans’ ‘English’
  - Exclusions: Reports on indication that was different to authorisation indication
- Surrogate endpoint categorisation according to Fleming & Power and Ciani *et al* hierarchies.

The protocol planning commenced in discussions between CSB, PB and PMcG on 22^nd^ January 2018. Over several weeks, the discussions developed the study focus, the research question outline and finally the literature search strategy which was discussed on 22nd February 2018. The research question grew from examination of the pivotal trials underpinning expedited approvals undertaken by JH, PB and PMcG during 2017.
